# *De Novo* Assembly and Annotation from Parental and F_1_ Puma Genomes of the Florida Panther Genetic Restoration Program

**DOI:** 10.1534/g3.119.400629

**Published:** 2019-09-13

**Authors:** Alexander Ochoa, David P. Onorato, Robert R. Fitak, Melody E. Roelke-Parker, Melanie Culver

**Affiliations:** * Department of Ecology, Evolution, and Organismal Biology, Ohio State University, Columbus, OH 43210,; † School of Natural Resources and the Environment, University of Arizona, Tucson, AZ 85721,; ‡ Fish and Wildlife Research Institute, Florida Fish and Wildlife Conservation Commission, Naples, FL 34114,; § Department of Biology, University of Central Florida, Orlando, FL 32816,; ** Leidos Biomedical Research, Inc., Frederick National Laboratory of Cancer Research, Bethesda, MD 20892, and; †† U.S. Geological Survey, Arizona Cooperative Fish and Wildlife Research Unit, Tucson, AZ 85721

**Keywords:** *Puma concolor*, Gene family expansion/contraction, Positive selection, Inbreeding depression, Genetic rescue

## Abstract

In the mid-1990s, the population size of Florida panthers became so small that many individuals manifested traits associated with inbreeding depression (*e.g.*, heart defects, cryptorchidism, high pathogen-parasite load). To mitigate these effects, pumas from Texas were introduced into South Florida to augment genetic variation in Florida panthers. In this study, we report a *de novo* puma genome assembly and annotation after resequencing 10 individual genomes from partial Florida-Texas-F_1_ trios. The final genome assembly consisted of ∼2.6 Gb and 20,561 functionally annotated protein-coding genes. Foremost, expanded gene families were associated with neuronal and embryological development, whereas contracted gene families were associated with olfactory receptors. Despite the latter, we characterized 17 positively selected genes related to the refinement of multiple sensory perceptions, most notably to visual capabilities. Furthermore, genes under positive selection were enriched for the targeting of proteins to the endoplasmic reticulum, degradation of mRNAs, and transcription of viral genomes. Nearly half (48.5%) of ∼6.2 million SNPs analyzed in the total sample set contained putative unique Texas alleles. Most of these alleles were likely inherited to subsequent F_1_ Florida panthers, as these individuals manifested a threefold increase in observed heterozygosity with respect to their immediate, canonical Florida panther predecessors. Demographic simulations were consistent with a recent colonization event in North America by a small number of founders from South America during the last glacial period. In conclusion, we provide an extensive set of genomic resources for pumas and elucidate the genomic effects of genetic rescue on this iconic conservation success story.

The puma (*Puma concolor*), also known as panther, mountain lion, or cougar, inhabits a broad range of ecosystems and has the widest range distribution of any terrestrial mammal in the Western Hemisphere (Sunquist and Sunquist 2002). Pumas share a recent common ancestor with jaguarundis (*P. yagouaroundi*) and cheetahs (*Acinonyx jubatus*) dating back to the Early Pliocene (Johnson *et al.* 2006; Ochoa *et al.* 2017). During the Late Pliocene, pumas colonized South America from the north as a result of the geological joining of the Panamanian land bridge (Webb and Rancy 1996). Reduced mtDNA and nuclear genetic variation in extant North American pumas support their having derived from a recent colonization event by a small number of founders from South America ca. 10,000−60,000 years before present (Culver *et al.* 2000; Ochoa *et al.* 2017).

Florida panthers represent the only remnant and viable puma population east of the Mississippi River (Nowell and Jackson 1996; Culver *et al.* 2000). In the mid-1900s, the Florida panther population was substantially reduced as a result of habitat loss and unregulated hunting, eventually persisting only in small habitat patches in South Florida (McBride *et al.* 2008; Onorato *et al.* 2010). By the early 1990s, inbreeding and decreased levels of genetic variation within the small population of < 30 adults resulted in several phenotypic traits characteristic of inbreeding depression, such as atrial septal defects, cryptorchidism, spermatozoal abnormalities, low testosterone levels, and high pathogen-parasite loads (Roelke *et al.* 1993a, 1993b; Barone *et al.* 1994; Cunningham *et al.* 1999; Johnson *et al.* 2010).

In the mid-1990s, eight female pumas from Texas were introduced into South Florida as part of a genetic restoration program implemented to reverse trends associated with inbreeding depression in Florida panthers (Seal and Lacy 1994; Johnson *et al.* 2010). Five of these females bred, producing at least 20 F_1_ offspring (Johnson *et al.* 2010; Onorato *et al.* 2010) that helped propel the increase in the Florida panther population size to 120‒230 individuals by 2018 (Florida Fish and Wildlife Conservation Commission 2018). Consequently, many of the phenotypic effects of inbreeding depression were mitigated (Hostetler *et al.* 2010; Johnson *et al.* 2010; Benson *et al.* 2011). For instance, examinations performed on juvenile and adult Florida panthers from 1990‒1995 revealed that 21% of individuals carried atrial septal defects and that 63% of males were cryptorchidic (Johnson *et al.* 2010). In contrast, only 7% of necropsied panthers from 2013‒2018 presented atrial septal defects and only 3% of necropsied males during that same period manifested undescended testes (Florida Fish and Wildlife Conservation Commission 2014, 2015, 2016, 2017, 2018).

In this study, we resequenced 10 genomes from partial Florida-Texas-F_1_ trios to assemble a consensus puma genome, examine the evolutionary and demographic history of this species, and reveal the genomic blueprint of the Florida panther genetic rescue program. Moreover, by performing functional annotations on an extensive set of coding genes, we provide paramount genomic resources that can be used as a reference for understanding the underlying molecular mechanisms involved in the expression of deleterious traits in Florida panthers.

## Materials and Methods

A complete description of the materials and methods—including supplemental tables, figures, and computer code—can be found in Supplemental Material.

### Sample collection and sequencing

We obtained whole blood samples—originally collected by Florida Fish and Wildlife Conservation Commission and National Park Service staff—from five female Texas pumas (TX101, TX105, TX106, TX107, and TX108), four male Florida panthers (FP16, FP45, FP60, and FP79), and one female Florida panther (FP73). Pedigrees and genetic ancestries for these individuals are summarized in Figure S1 (also see Johnson *et al.* 2010 and Ochoa *et al.* 2017).

Briefly, Texas pumas TX101, TX105, TX106, and TX108 and Florida panthers FP16, FP45, FP60, and CM7 (unsampled male) constitute the known founder individuals to viable F_1_ panthers that resulted from the mid-1990s genetic restoration program. TX101 and CM7 produced two F_1_ offspring: FP73 and FP79. The latter, FP79, would then breed with TX107. Specifically, FP45, FP60, and CM7 were ‘pure’ or canonical Florida panthers from Big Cypress National Preserve; FP16 was a non-canonical Florida panther with Costa Rican and Panamanian ancestry from the Everglades National Park.

We isolated genomic DNA using a phenol-chloroform extraction protocol and prepared paired-end (PE) libraries of 500-bp inserts from each sample and a mate-pair (MP) library of 5-kb inserts from sample FP16. We sequenced each library on independent lanes of an Illumina HiSeq 2000/2500 system (Illumina Inc., San Diego, California) at the University of Arizona Genetics Core (UAGC, Tucson, Arizona).

### De novo assembly and annotation

We used Trimmomatic v.0.35 (Bolger *et al.* 2014) to remove adapter sequences from the PE and MP reads and to trim them based on quality. We error-corrected the PE reads using Musket v.1.1 (Liu *et al.* 2013). We assembled the trimmed and error-corrected PE reads in ABySS v.1.3.6 (Simpson *et al.* 2009) using *k*-mer lengths of *k* = 45, 51, 55, 59, 61, 63, 65, 69, and 75 bp. We used the trimmed MP reads to arrange the resulting contigs into scaffolds; ultimately, we retained only scaffolds ≥ 500 bp. Using CEGMA v.2.4 (Parra *et al.* 2007), we identified the presence of mammalian core eukaryotic genes (CEGs).

We annotated repetitive elements and structural RNAs from the best assembly using RepeatMasker v.4.0.7 (Smit *et al.* 1996–2010) and Infernal v.1.1.2 (Nawrocki and Eddy 2013), respectively. We performed gene annotations with MAKER2 v.2.31.6 (Cantarel *et al.* 2008; Holt and Yandell 2011) using an iterative procedure that included i) gene models built from the identified CEGs using SNAP (Korf 2004); ii) *ab initio* predictions from GeneMark-ES (Lomsadze *et al.* 2005) and AUGUSTUS v.2.5.5 (Stanke *et al.* 2006); iii) transcriptome sequences from the puma (Fitak *et al.* 2016); and iv) protein sequences from the cheetah, cat, tiger, and dog downloaded from Ensembl (www.ensembl.org). We selected gene predictions with an exon annotation edit distance (eAED) < 0.75 (Holt and Yandell 2011). We annotated the longest isoform from each gene prediction using Blast2GO v.4.0.7 (Conesa *et al.* 2005).

### Interspecific comparative analyses

We used OrthoMCL v.1.0 (Li *et al.* 2003) to define protein homologs (*i.e.*, orthologs and paralogs) across the puma, cat, dog, panda, cow, human, and mouse. We clustered proteins into gene families by performing all-against-all local alignments. We used DupliPHY v.1.0 (Ames *et al.* 2012; Ames and Lovell 2015) to identify gene family expansion and contraction events based on divergence times derived from www.timetree.org and Ochoa *et al.* (2017). We excluded gene families absent in either the puma, cat, dog, panda, and cow clade or the mouse and human clade.

To detect positive selection, we aligned each single-copy ortholog across species with PRANK v.170427 (Löytynoja and Goldman 2005). We used trimAl v.1.4 (Capella-Gutiérrez *et al.* 2009) to eliminate poorly aligned amino acid residues and to convert the resulting clean alignment to the corresponding codon (*i.e.*, nucleic acid) alignment. We conducted likelihood ratio tests with PAML v.4.9 (Yang 1997, 2007) by comparing the M1a branch model, in which lineages evolve neutrally, with the M2a branch model, in which we assumed that the puma lineage evolved under positive selection. We computed the *P*-values from each likelihood ratio test using the χ^2^ statistic adjusted by the false discovery rate method (Benjamini and Hochberg 1995).

### Intraspecific comparative analyses

We mapped the PE reads from each puma sample to the reference assembly using BWA v.0.7.9 (Li and Durbin 2009). We used GATK v.3.8.0 (McKenna *et al.* 2010) and VCFtools v.0.1.12 (Danecek *et al.* 2011) to identify and validate SNPs across samples. We retained SNPs with genotypes present across each of the following groups: FL (samples FP45 and FP60), TX (samples TX101, TX105, TX106, TX107, and TX108), F_1_ (samples FP73 and FP79), and sample FP16.

We examined historical changes in effective population sizes, *N_e_*, with PSMC v.0.6.4 (Li and Durbin 2011) using 100 bootstrap replicates for each run. We scaled the final *N_e_* estimates to a generation time of three years and a mutation rate of 6.6×10^−9^ substitutions site^−1^ generation^−1^ (Kumar and Subramanian 2002).

### Data availability

All raw sequencing data have been archived in GenBank and can be accessed through BioProject accession number PRJNA422772 (in particular, SRA run numbers SRX3557019–29). The puma genome assembly is available in GenBank as accession PSOM00000000. Supplemental Material, File S1, and additional input/output data for replicating the analyses have been deposited in Figshare: https://doi.org/10.25387/g3.9820661.

## Results and Discussion

We generated ∼1.3 billion PE reads from 10 pumas and ∼136 million MP reads from a single individual, of which 90.9% and 44.7%, respectively, were retained after quality control procedures (Table S1; Figure S1). Our final assembly was ∼2.6 Gb in length, consisted of 152,283 scaffolds with an N50 of 193,863 bp, and contained 96.4% of CEGs (Figure S2); the overall coverage of this assembly was ∼74×. Repetitive regions accounted for 29.2% of the puma genome (Table S2) and 3931 structural RNAs from 636 families were identified (Table S3).

We defined 22,745 protein-coding genes and functionally annotated 20,561 (90.4%) with Blast2GO (Table S4; Figures S3–S8; File S1). Based on these annotations, we identified ∼1100 genes related to atrial cardiac development, testicular and spermatozoal morphogenesis, testosterone synthesis and regulation, and immune response (File S1), all of which could be further explored for potential relationships with the expression of deleterious traits observed in the Florida panther.

**Figure 1:**
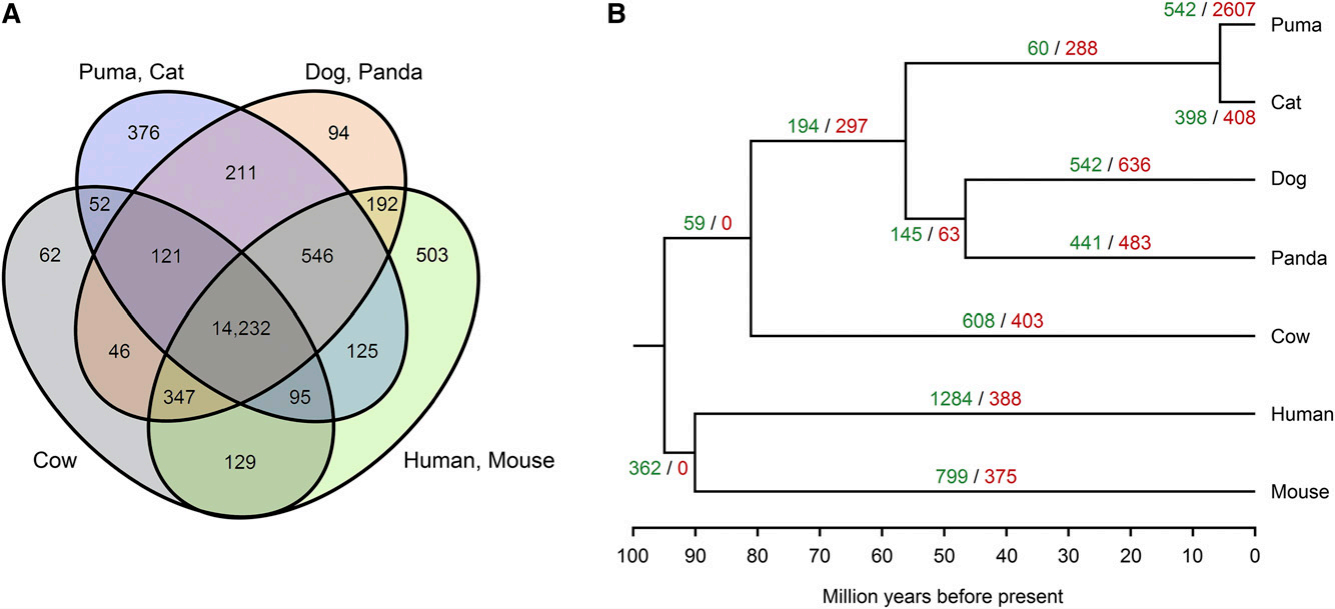
Interspecific gene family features. (A) Venn diagram representing unique and shared gene families within and among the puma, cat, dog, panda, cow, human, and mouse. (B) Phylogenetic tree showing the number of gene family expansions (green) and contractions (red) at each branch across species.

We characterized 17,131 gene families across the puma, cat, dog, panda, cow, human, and mouse genomes (Figure 1A). Of these, pumas presented 542 gene family expansions and 2607 contractions with respect to the puma-cat most recent common ancestor (Figure 1B). Biological processes enriched in the expanded gene families included neuronal and embryological development, determination of adult lifespan, and binding of sperm to zona pellucida (Table S5). For example, the *PLXN* gene family, which contains 11 paralogs, is necessary for the signaling of semaphorins on the surface of axons and for the subsequent remodeling of the cytoskeleton; it also supports invasive growth and cell migration in the hippocampus for spatial memory enabling orientation and navigation (Cheng *et al.* 2001; Suto *et al.* 2005; Ben-Zvi *et al.* 2008; He *et al.* 2009).

Among the contracted gene families, 39 were associated with the sensory perception of smell (Table S6) and represented a larger family of G protein-coupled olfactory receptors, *ORs*, coded by single-exon genes (Young *et al.* 2002; Zhang and Firestein 2002; Malnic *et al.* 2004). This finding represents an unexpected result considering that genomes of other feline species, particularly the tiger and cheetah, are enriched for olfactory and G protein-coupled receptor activity (Cho *et al.* 2013; Dobrynin *et al.* 2015). Other contracted gene families were associated with long-chain fatty acid synthesis, such as arachidonate, and with carboxylic acid transport. Since lipid metabolism is essential for digestion in obligate carnivores and for reproduction (Irizarry *et al.* 2012; Cho *et al.* 2013), pumas could be using alternate, or otherwise undescribed, pathways to generate and transport these compounds, as noted in the domestic cat (Montague *et al.* 2014).

Analysis of 8210 single-copy orthologs found evidence of positive selection in 512 genes adjusted *P* < 0.05 in all cases. The latter were enriched for biological processes associated with the targeting of proteins to the endoplasmic reticulum, the degradation of mRNAs with intermediate stop codons, and the transcription of viral genomes within host cells (Table S7). Furthermore, we hypothesize that in pumas, loss of *OR* genes could be coupled with the refinement of multiple sensory capabilities—including the sense of smell—as similar tradeoffs have been documented in the domestic cat and other mammals (Gilad *et al.* 2004; Warren *et al.* 2008; Montague *et al.* 2014). To this extent, we detected 17 genes related to different senses (most notably to vision; Table S8) that could have been positively selected in response to species-specific nocturnal activity, hunting, and sociochemical communication (Harmsen *et al.* 2011; Allen *et al.* 2016; Pratas-Santiago *et al.* 2017).

**Figure 2:**
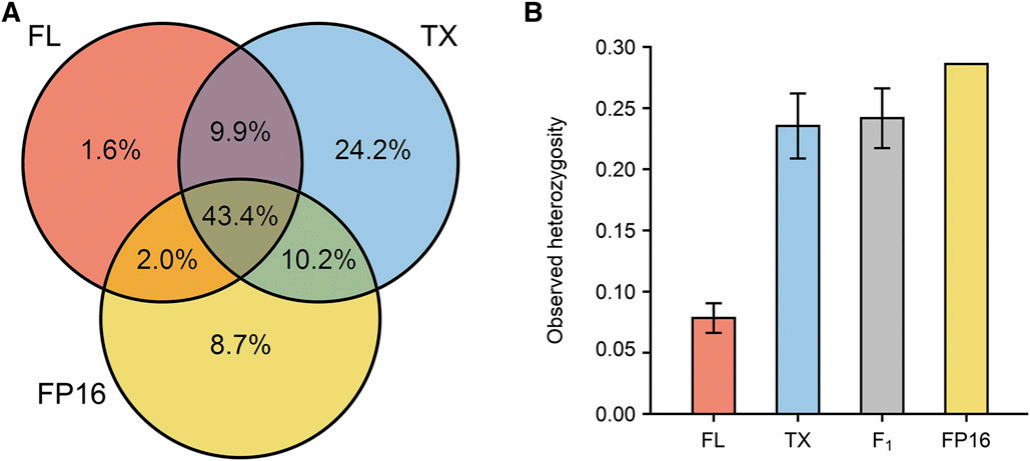
Intraspecific genetic variation. (A) Distribution of variants across 6,210,080 biallelic SNPs in three lineages: FL (canonical Florida panthers FP45 and FP60), TX (Texas pumas TX101, TX105, TX106, TX107, and TX108), and FP16 (a non-canonical Florida panther with Costa Rican and Panamanian ancestry). The proportion of unique and shared alleles within and among lineages is indicated inside each circle. Unique alleles found in the F_1_ Florida panthers FP73 and FP79 were assumed to have derived from another canonical Florida panther, CM7, which was not sampled. (B) Mean observed heterozygosity in the FL, TX, F_1_, and FP16 sample groups. The number of heterozygous genotypes per individual was scaled to the total number of validated genotypes for that sample in the subset of 6,210,080 sites (Table S10). Standard deviations for multisample groups are shown as whiskers on each bar.

We validated 6,210,080 biallelic SNPs (Ti/Tv = 2.228) across the entire sample set after mapping ∼1 billion processed PE reads (Table S9). Of these SNPs, only 1.6% of alleles were exclusive to canonical Florida panthers (samples FP45, FP60, and, partially, CM7) and 8.7% of alleles were exclusive to a non-canonical Florida panther of Costa Rican and Panamanian ancestry (sample FP16) (Figures 2A and S1; O’Brien *et al.* 1990; O’Brien and Roelke 1990; Roelke *et al.* 1993a; Culver *et al.* 2000; Ochoa *et al.* 2017). However, the proportion of private alleles present in canonical Florida panthers (*i.e.*, 1.6%) is likely underestimated because an undetermined fraction of alleles from sample FP16 are also of canonical Florida panther origin, and because we analyzed a reduced—albeit highly inbred—collection of Florida panthers.

Of the aforementioned SNP dataset, 24.2% of alleles were exclusive to Texas pumas (samples TX101, TX105, TX106, TX107, and TX108) (Figure 2A). Therefore, in as many as 48.5% of the polymorphic sites examined, Texas pumas could have contributed novel alleles to the Florida panther gene pool. These proportions, nevertheless, are likely to decrease, since fractions of unique Texas alleles—as defined in this study—could have been represented in the mid-1990s Florida panther population prior to admixture with Texas pumas and/or may not have been inherited to the subsequent Florida panther generation. Despite such uncertainties, there is little doubt that F_1_ Florida panthers, namely samples FP73 and FP79, experienced a threefold increase in observed heterozygosity (Figure 2B; Table S10) with respect to their immediate, canonical Florida panther predecessors as a result of the introduction of the Texas pumas.

Simulations with PSMC (Figure S9) indicated that canonical Florida panther and Texas puma lineages reached a maximum *N_e_* of ∼40,000−60,000 individuals during the Late Pleistocene (ca. 30,000−60,000 years before present), after which a sharp decline in *N_e_* was observed. This result is consistent with a recent founder effect and colonization event in North America by individuals from South America during the last glacial period (Culver *et al.* 2000; Ochoa *et al.* 2017).
